# Effects of periadolescent fluoxetine and paroxetine on elevated plus-maze, acoustic startle, and swimming immobility in rats while on and off-drug

**DOI:** 10.1186/1744-9081-7-41

**Published:** 2011-10-05

**Authors:** Charles V Vorhees, LaRonda R Morford, Devon L Graham, Matthew R Skelton, Michael T Williams

**Affiliations:** 1 Division of Neurology, Department of Pediatrics, Cincinnati Children's Research Foundation and University of Cincinnati College of Medicine, 3333 Burnet Ave. Cincinnati, OH, 45229, USA; 2 Eli Lilly and Company, Greenfield, IN, 46140 USA

**Keywords:** fluoxetine, paroxetine, acoustic startle response, elevated plus maze, forced swim test, corticosterone, adolescent brain development

## Abstract

**Rationale:**

Whether selective serotonin reuptake inhibitors (SSRIs) exposure during adolescent brain development causes lasting effects remains unresolved.

**Objective:**

Assess the effects of fluoxetine and paroxetine 60 days after adolescent exposure compared with when on-drug.

**Methods:**

Male Sprague-Dawley littermates (41 litters) were gavaged on postnatal days 33-53 with fluoxetine (3 or 10 mg/kg/day), paroxetine (3, 10 or, 17 mg/kg/day), or water; half were tested while on-drug (21 litters) and half after 60 days off-drug (20 litters).

**Results:**

The highest dose of the drugs reduced body weight gain during treatment that rebounded 1 week post-treatment. On-drug, no significant group differences were found on elevated plus maze time-in-open, zone entries, or latency to first open entry; however, the high dose of paroxetine significantly reduced head-dips (N = 20/group). No significant effects were found on-drug for acoustic startle response/prepulse inhibition (ASR/PPI) although a trend (p < 0.10) was seen, which after combining dose levels, showed a significant increase in ASR amplitude for both fluoxetine and paroxetine (N = 20-21/group). No differences on immobility time were seen in the Porsolt forced swim test or in plasma corticosterone at the end of forced swim (N-19-21/group). Off-drug, no effects were seen in the elevated plus maze (N = 16/group), ASR/PPI (N = 20/group), forced swim (N = 19-20/group), or plasma corticosterone (N = 19/group). At the doses tested, fluoxetine and paroxetine induced minor effects with drug on-board but no residual, long-term adverse effects in rats 60 days after drug discontinuation.

**Conclusions:**

The data provide no evidence that fluoxetine or paroxetine have long-term adverse effects on the behaviors measured here after adolescent to young adult exposure.

## Background

Selective serotonin reuptake inhibitors (SSRIs) are widely used antidepressants that inhibit the reuptake of serotonin (5-HT) into the presynaptic terminal by binding to the serotonin transporter (SERT). SSRIs have been used with success in adults for treatment of depression, premenstrual syndrome, obsessive compulsive behavior, panic disorder, generalized anxiety disorder, social anxiety disorder, and post-traumatic stress disorder [1-3]. A meta-analysis of adult trials indicates that suicidality may be increased shortly after antidepressant treatment is begun regardless of whether the drug is an SSRI or tricyclic antidepressant [4].

SSRIs have also been used in children and adolescents, primarily for depression [5]. It has been suggested that children and adolescents may be at higher risk of adverse effects perhaps because SSRIs are contraindicated for some types of depression (such as bipolar disorder) that are often unrecognized in younger patients [5]. A recent meta-analysis of randomized controlled trials of SSRI treatment for depression in children and adolescents found benefit from SSRI treatment, but not all SSRIs were equally effective. Fluoxetine, sertraline, and citalopram were most effective in this age group compared with other SSRIs [6].

Both efficacy and adverse effects of SSRIs in younger patients are ongoing areas of investigation. Modeling the effects of juvenile SSRI treatment in rats [7,8] and mice [9] demonstrated that between postnatal (P) day 21 and 35, there are differences in drug response [10]. However, data on SSRI exposure during adolescence in rodents has received less attention. There are two issues concerning age-related effects of SSRI exposure: (1) efficacy and (2) long-term or adverse effects.

In one experiment, male Wistar rats were treated with 15 mg/kg of paroxetine or 30 mg/kg of fluvoxamine by gavage from postnatal days (P) 33-62 and tested 20-21 days later for sexual behavior, 23-24 days later in the elevated plus-maze (EPM), 31 days later for acoustic startle response (ASR) with prepulse inhibition (PPI), 35-36 days later in the Porsolt forced swim test (FST), and 56 days later in an elevated T-maze [11]. Both drugs decreased time in open arms in the EPM, but in an elevated T-maze in which 5 trials were given from a closed stem to open arms, only on trial-2 did fluvoxamine significantly increase time to enter an open arm. On the converse arrangement (open to closed entry), no change was seen. No change in ASR/PPI, immobility in the FST, or in 8-OH-DPAT-induced 5-HT_1A_-related behaviors was observed (lower lip retraction or sexual facilitation). A reduction in ejaculatory frequency was found in the drug groups during the third week of testing, but not during weeks 1 and 2; no differences were found for ejaculatory latency, mount frequency, or intromissions.

More recently the effects of fluoxetine were tested in adolescent mice [12]. C57BL/6J and BALB/cJ male mice were treated with fluoxetine in drinking water at concentrations of 80 or 160 mg/L on P21-42 or P56-84 with testing 30 days later on open-field, EPM, cued conditioned fear and extinction, the FST, plasma corticosterone, and brain 5-HT and 5-HIAA. The lower dose was calculated to be 9-10 mg/kg/day and the higher dose 17-18 mg/kg/day. In neither strain, at neither dose did significant effects emerge [12]. No changes in 5-HT, 5-HIAA, or corticosterone after the FST were seen.

Hence, the evidence is divided as to whether SSRI exposure during adolescent brain development has immediate and/or untoward lasting effects. The purpose of the present experiment was to test whether adolescent exposure to two widely used SSRIs (fluoxetine and paroxetine) has immediate or long-term effects. The previous experiments tested animals 21-56 or 30 days after drug exposure. To determine if effects occur while on-drug and persist after drug cessation, we treated two cohorts of rats identically and tested one cohort while on-drug and the other after drug discontinuation.

We used the Porsolt FST because it is related to depression, the therapeutic target of these drugs. We assessed sensorimotor gating using ASR/PPI because this is a widely used marker for abnormalities in neuropsychiatric disorders [13-16]. We used the EPM to determine whether SSRIs cause changes in anxiety, and we assessed plasma corticosterone after FST to determine if stress reactivity was altered by SSRI exposure.

There are many SSRIs. We focused on paroxetine because it showed residual effects previously [11] and fluoxetine because it is FDA approved for use in juvenile and adolescent patients (6-18 years of age). We used the same treatment days as de Jong et al. [11], i.e., P33-62, an age range from puberty to young adulthood in rats. One cohort was tested while on-drug after 25 days of prior treatment (tested on P57-62) and the other cohort off-drug (P122-127; 60 days post-treatment). We tested doses from the literature showing effects after adolescent exposure [11,12]. A third dose of paroxetine was included to match the dose used by de Jong et al. [11].

## Methods and materials

### Animals and treatments

Male (251-275 g) and female (151-175 g) Sprague-Dawley CD, IGS rats (Charles River, Raleigh, NC) were bred in house after one to four weeks of acclimation to the vivarium and offspring were the subjects for these experiments. Evidence of pregnancy was designated as E0 (embryonic day zero) and most females delivered on E22. Birth was designated P0 and litters were culled to 6 males and 2 females on P1. Litters not having a sufficient number of males were not included in the experiment. At total of 41 litters were enrolled in the protocol. All litters were treated identically, the only difference being that offspring from 21 litters were tested while on drug (Group A testing began on P57) and offspring from the remaining 20 litters were tested beginning 60 days after the last day of treatment (Group B testing began on P122). Only males were tested, therefore, 6 animals/litter × 41 litters = 246 animals in the experiment. Animals were maintained in polycarbonate shoebox cages (46 × 24 × 20 cm) on a 14 h light/10 h dark cycle (lights on at 600 h) with food (NIH-07 diet) and water available ad lib in a vivarium maintained at 21 ± 1°C with 50 ± 10% humidity and accredited by the Association for the Accreditation and Assessment of Laboratory Animal Care (AAALAC). The research protocol was approved by the Cincinnati Children's Research Foundation's Institutional Animal Care and Use Committee. Offspring were separated from their dams on P28 and housed 2 per cage for the remainder of the experiment. Each cage contained a semicircular stainless steel enclosure (17.8 cm long with the open side 18.4 cm across and 10.2 cm high with the open side down made of 16-gauge stainless steel) as an additional environmental enrichment.

### Drugs and doses

Animals were weighed daily during treatment and weekly thereafter. All animals received a daily gavage from P33-62 of 5 ml/kg body weight of USP grade water alone or water containing one of the drugs. Drugs were obtained as hydrochloride salts and all doses are expressed as the salt form. The treatment groups were as follows: Control (water), Flu3 (Fluoxetine 3 mg/kg), Flu10 (fluoxetine 10 mg/kg), Par 3 (paroxetine 3 mg/kg), Par10 (paroxetine 10 mg/kg), and Par 17 (paroxetine 17.04; this dose matched the dose of paroxetine reported previously [11] as 15 mg/kg expressed as the freebase). Fluoxetine HCl was obtained from Eli Lilly and Company (Indianapolis, IN) and paroxetine HCl was obtained from Suzhou ChonTech PharmaChem Technology Company (Suzhou, China).

### Drug stability and potency

Drugs were mixed weekly in 5 ml of water in the appropriate concentrations to deliver the specified doses. In order to verify drug concentrations for each solution, a set of drug solutions at each concentration was made and sent to ABC Laboratories (Columbia, MO) for verification. In order to ensure stability of these solutions a set of the highest concentration solution of each drug was made and analyzed at 0, 3, or 7 days post-mixing by ABC Laboratories. Finally, drug potency was assayed on both drug stocks at the end of the experiment to ensure that no degradation had occurred by sending an aliquot of each drug to ABC Laboratories for analysis.

Drug concentrations were analyzed via high performance liquid chromatography with ultraviolet detection (HPLC-UV) at 227 nm. Samples were injected onto a Zorbas SB-Phenyl reversed phase column (250 × 4.6 mm, 5 μm). The HPLC system was an Agilent HP 1100. The mobile phase was 50% acetonitrile, 50% 0.1% TFA aqueous. The flow rate was 1.0 ml/min, attenuation was 1000 mAU at ambient temperature with a column temperature of 30°C. Sample size was 20 μl and run time was 10 min. Final drug concentrations were calculated from a standard curve in the linear range.

### Behavioral testing and sample collection

Group A and B animals were tested in the same behavioral tasks but at different ages. Group A had 21 litters and Group B had 20 litters resulting N/group for each test ranging from 19-21/test for Group A and 16-20/test for Group B. The tests were (in order): EPM, ASR/PPI, and the Porsolt FST (immediately followed by decapitation and blood collection for determination of plasma corticosterone). There were 2 days of separation between each test. Group A was tested in the EPM on P57, ASR/PPI on P59, and FST on P61-62. Group B was tested in the EPM on P122, ASR/PPI on P124, and FST on P126-127. For Group A, all tests while on-drug were given not less than 1 h and not more than 4 h after each daily dose.

### Elevated plus maze

This test was conducted on a single day. The apparatus was constructed of high-density, black polyethylene polymer. Each arm was 10 × 50 cm, with two opposing open arms and two perpendicular opposing arms with high side walls (closed arms). The walls were 50 cm high in the closed arms and the open arms had 0.6 cm curbs on the edges to prevent falls. The apparatus was mounted 50 cm above the floor. During testing the room was lit by a single dimmed halogen lamp (aimed upward to reflect off the ceiling). Each animal was tested for 5 min and movements were recorded on a DVD recorder using an overhead camera. Behavior was later played back and scored by an observer blind to treatment group for time-in-open, number of open entries, latency to first open entry, and head dips. An arm was scored as entered if the animal's head and two front feet crossed a boundary line between the center region and that arm.

### Acoustic startle/PPI

This test was conducted on a single day. Acoustic startle reactivity with reflex modified inhibition by prepulse stimulation (ASR-PPI) was measured in four identical SR Lab test chambers (San Diego Instruments, San Diego, CA). Each test chamber was calibrated using the manufacturer's guidelines and sensitivity was regularly checked using a calibrated oscillation device to ensure consistent sensitive readings. At the start of each test, animals were placed in acrylic cylindrical tubes mounted atop piezoelectric force transducers positioned inside sound attenuating test chambers. Background white noise was set at 70 dB. The test paradigm reported previously was duplicated as closely as possible [11]. Each test session consisted of a 5 min acclimation period followed by a 5 × 5 Latin square sequence of trials of 5 different types: no stimulus, startle signal with no prepulse, startle signal with prepulse 3 dB above background (73 dB), startle signal with prepulse 5 dB above background (75 dB), and startle signal with prepulse 10 dB above background (80 dB). Each animal received each trial type once in each of the 5 orders, and the entire Latin square sequence was repeated; hence, each animal received each trial type/order twice. Trials of the same type were averaged together for analysis. The intertrial intervals were 10-20 s with randomized spacing. The interstimulus interval was 100 ms (measured from prepulse onset to startle signal onset). The startle signal was a 120 dB mixed frequency, white noise burst that lasted for 20 ms. The recording window was 100 ms. Prepulses lasted for 20 ms. Stimulus intensity was measured using a Quest sound level meter (SPL scale) with the meter placed in the test chamber in the center of the test stage with the door closed and the microphone directed upward toward the ceiling-mounted speaker. Response amplitude (V_max _= maximum voltage change within the recording window) was recorded in units of voltage change (mV). Test chambers were cleaned with 70% ethanol between animals.

### Porsolt forced swim test

Testing was conducted over two successive days using the original method of Porsolt for rats [17,18]. The apparatus consisted of four clear acrylic cylinders 19 cm in diameter (i.d.) and 60 cm in height filled to a depth of 30 cm (to avoid water depth concerns [19]) with room temperature water (21-23°C). On day-1 rats were placed in the cylinders for 15 min and on day-2 for 5 min. Each chamber was separated by black acrylic partitions to prevent animals from seeing one another. Movement was recorded using a video camera and DVD recorder. Scoring was performed by an experimenter blind with respect to treatment group. Immobility duration was scored on day-2 as time spent not swimming or reaching at the walls. Occasional, minor limb movements were permitted in order for the animal to maintain balance, brace itself against the wall, or tread water to keep afloat so long as a coordinated multi-limb swimming motion was not present. We did not use the expanded FST method [20,21] because we were not testing SSRI efficacy, but rather for toxicity.

### Tissue collection

Upon the completion of the FST, animals were removed from the water, carried to another room where they were decapitated (< 30 s from removal from FST), and blood collected for later plasma corticosterone determinations.

### Corticosterone assay

Blood was collected in tubes that contained EDTA (2% in 0.05 ml). Samples were centrifuged for 15 min at 4°C, plasma removed and stored at -80°C until assayed. Corticosterone concentrations were determined by diluting plasma 1:10 in assay buffer and assayed in duplicate using a commercially available EIA kit (IDS, Fountain Hills, AZ). All determinations were made from kits originating from the same lot.

### Statistical analyses

Data for Group A and Group B animals were analyzed separately. Data were analyzed using completely randomized block analyses of variance (ANOVA) mixed linear models (SAS Proc Mixed, SAS Institute, Cary, NC). The Kenward-Roger adjusted degrees of freedom method was used and can be fractional. In these analyses, litter was the block factor. Data from the EPM and FST had 1-between-subject factor (treatment group). ASR-PPI had 1-between-subject factor (treatment group) and 1-within-subject factor (trial). For body weight, the ANOVA has 1-between-subject factor (treatment group) and 1-within-subject factor (age). Interactions were analyzed using the slice-effect ANOVA method on each level of within-subject factors. *A posteriori *group comparisons were analyzed using the Hochberg step-up method to control for multiple comparisons. As follow-up we report effect size (ES) for drug treatment for each behavior. There are no methods for calculating ES for mixed linear ANOVA, therefore, we reanalyzed the data using general linear model ANOVA (GLM) again with litter as a random block factor. SAS GLM provides three indices of ES (noncentrality, partial, and semipartial correlation ratios). These reanalyses produced results nearly identical to those obtained from Mixed model analyses, thereby validating the use of ES values from GLM ANOVA models. We then took the semipartial correlation ratios (eta-square) and converted them to Cohen's f (similar to Cohen's d, except for ANOVAs rather than for t-a test) and followed Cohen's categorization scheme (small ES are around 0.2, medium around 0.5, and large ≥ 0.8 [22]. Significance was taken as p ≤ 0.05 and data are presented as least square mean ± SEM.

## Results

### Drug Concentrations and Stability

Drug solutions of both compounds were prepared and sent to ABC Laboratories for analytical chemistry to determine drug concentrations by HPLC analysis (Methods; M-1736-000 and USP 30 Paroxetine HCl). Each sample was analyzed in triplicate. The first set of samples was taken from prepared test solutions for concentration verification. Mean concentrations of the test solutions were within 3% of target concentrations.

Stability Assessment: Fluoxetine hydrochloride is known to be stable in solution ≥ 7 days but to determine the stability of paroxetine we tested it at the low and high concentrations at three time points: immediately after preparation and at 3 and 7 days. The paroxetine solutions showed no decrease in active drug content over the 7-day period.

End of Study Drug Purity: After the completion of the experiment, crystalline samples of both drugs were sent to ABC Laboratories to determine drug purity. Both paroxetine HCl and fluoxetine HCl were unchanged (mean percent potency of 101%).

### General Characteristics

There were no deaths; 100% of the animals enrolled in the experiment survived and were tested. Group sizes varied slightly from the design because of experimenter error or equipment malfunction. The exact N for each test is provided in the figure captions.

Body weight data were analyzed by age. Once offspring were assigned to treatment groups, their body weights from P1-28 were analyzed to ensure that no preexisting differences occurred. This analysis showed no differences as a function of later assignment to the groups (F(5,200) = 1.09, p > 0.36). The effect of age was significant (F(4,960) = 23327.9, p < 0.0001), demonstrating time-dependent growth. The group × week effect was not significant (F(20,960) = 0.50, p > 0.96).

**Figure 1:**
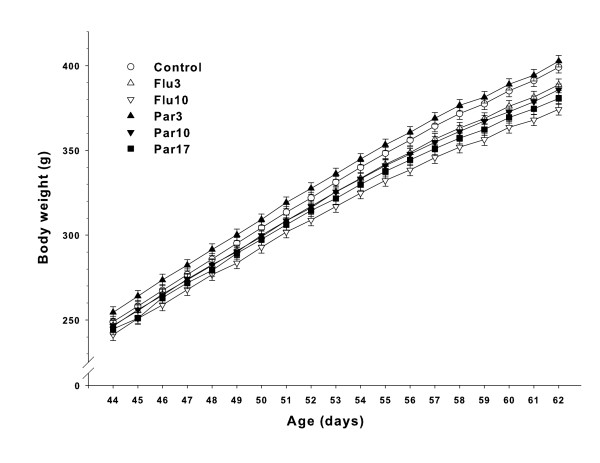
**Body weights during treatment: Data represent least square mean (± SEM) body weights (g) on days on which significant group differences were obtained**. Treatment was from P33-62; significant group differences occurred on P44-62. Groups A and B are combined. Group sizes: Group A = 21, Group B = 20.

**Figure 2:**
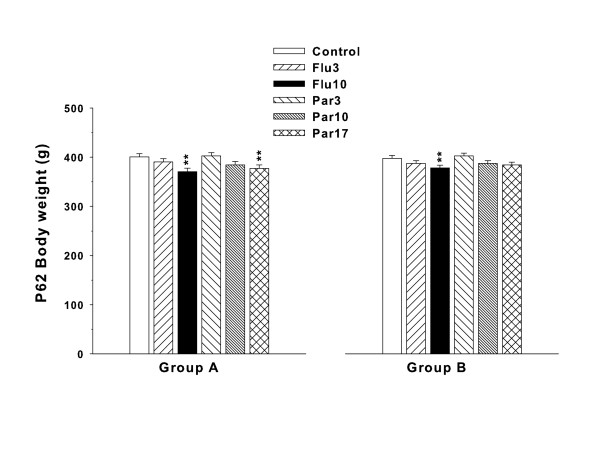
**Body weights: Least square Mean (± SEM) body weights on the last day of treatment (P62)**. Group A and B are shown separately. There were 21 litters in Group A and 20 litters in Group B. **p < 0.01 vs. Control.

Body weights during drug treatment (P33-62) were analyzed with Groups A and B combined. There was a significant effect of treatment (F(5,227) = 4.84, p = 0.0003), day (F(29,6922) = 1481.86, p < 0.0001), and treatment × day (F(145,6931) = 1.49, p = 0.0001). Slice-effect ANOVAs on each day showed no treatment effects on P33-43, however significant effects were obtained on days P44-62. Since there were no significant group differences before P44, only the P44-62 body weight data are illustrated in Figure 1. Body weights on the last day of treatment (P62) were analyzed separately for Groups A and B and are shown in Figure 2. As can be seen, for Group A, the Flu10 and Par 17 groups weighed less than Control. For Group B, only the Flu10 group weighed less than Control. The Par17 for Group B was not significantly different from Control (p = 0.12).

**Figure 3:**
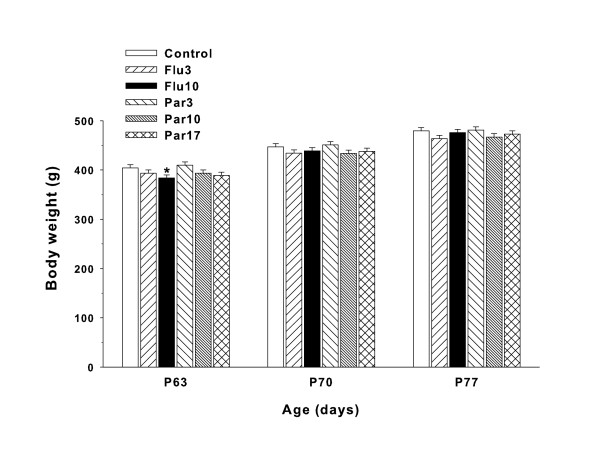
**Body weights: Least square Mean (± SEM) body weights for Group B during the two weeks after the end of treatment**. Significant group differences occurred on P63; only the Flu10 group weighed significantly less than Controls. From P70 to the end of the experiment, there were no significant treatment group differences. *p < 0.05.

Group A animals completed testing by P62, hence there are no body weight data for this group after P62. For Group B, body weights were recorded on P63 and weekly thereafter until the end of testing on P126. In order to map body weight recovery after the end of treatment, data for the first two weeks after the end of treatment (P63-77) were analyzed separately. There was no significant treatment main effect (F(5,95) = 1.94, p < 0.10). The day/week factor in the analysis was significant (F(2,228) = 4256.37 = p < 0.0001), indicating time-dependent growth, and there was a significant treatment × week interaction (F(10,228) = 9.59, p < 0.0001). Slice-effect ANOVAs on each week showed differences on P63 (p < 0.01) but not on P70 or P77, indicating that recovery was rapid (< 1 week). This pattern is illustrated in Figure 3. As can be seen, only the Flu10 group weighed significantly less on P63 and no differences remained on P70, P77, or thereafter (i.e., between P84-126 (not shown)).

### Elevated plus maze

**Figure 4:**
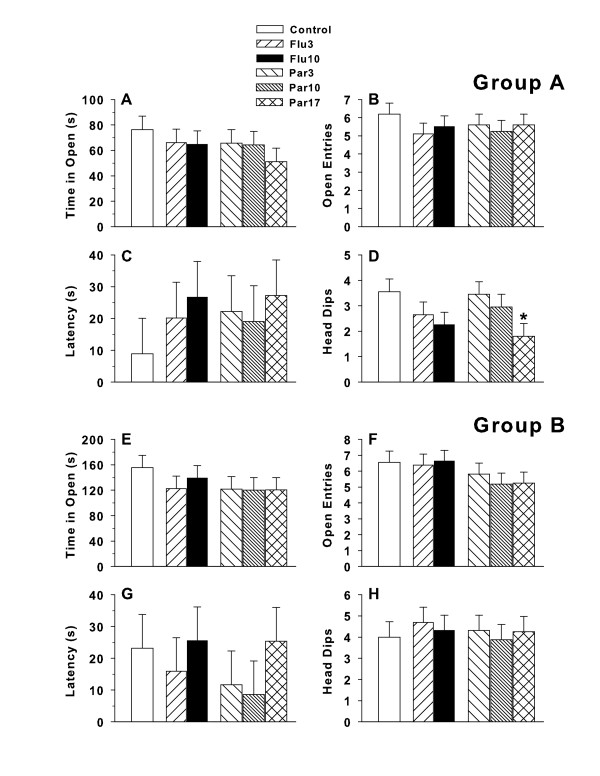
**Elevated plus maze: Data shown are least square means (± SEM) on the EPM during the 5 min test session**. ***A,E***: time (s) spent in open arms; ***B,F***: number of open arm entries; ***C,G***: latency to first open arm entry; and ***D,H***: number of open arm head dips. Group A was tested on P57 and Group B on P122. Group sizes for Group A: Number of litters tested = 21; data are based on the following number of subjects/group: Control = 20, Flu3 = 20, Flu10 = 20, Par3 = 20, Par10 = 20, Par17 = 20. One litter's data were missing because of a DVD playback defect. For Group B: Number of litters tested = 20; data are based on the following number of subjects/group: Control = 16, Flu3 = 16, Flu10 = 16, Par3 = 16, Par10 = 16, Par17 = 16. Data were unrecoverable from 4 litters whose recorded data tracks would not playback from the DVD.

For Group A there were no significant differences in time spent in the open arms, number of open arm entries, or latency to first open arm entry (Figure 4A-C, **respectively**). A significant treatment group effect was found on number of head dips (F(5,95) = 2.74, p < 0.03). Post hoc group comparisons showed that the Par17 group had fewer head dips than Controls (Figure 4D). No other group comparisons were significant. ES for time in open arms were = 0.15 (small), for arm entries = 0.13 (small), for latency = 0.12 (small), and for head dips = 0.27 (small).

For Group B there were no significant differences in time spent in the open arms, number of open arm entries, latency to first open arm entry, or head dips (Figure 4E-H, **respectively**). ES for time in open arms = 0.18 (small), for arm entries = 0.22 (small), for latency = 0.16 (small), and for head dips = 0.09 (small).

### Acoustic Startle/PPI

ASR-PPI data were analyzed two ways: (a) by prepulse intensity, and (b) using the 0 prepulse response amplitude as a covariate by ANCOVA in order to assess PPI after controlling for any possible differences in basal startle reactivity.

**Figure 5:**
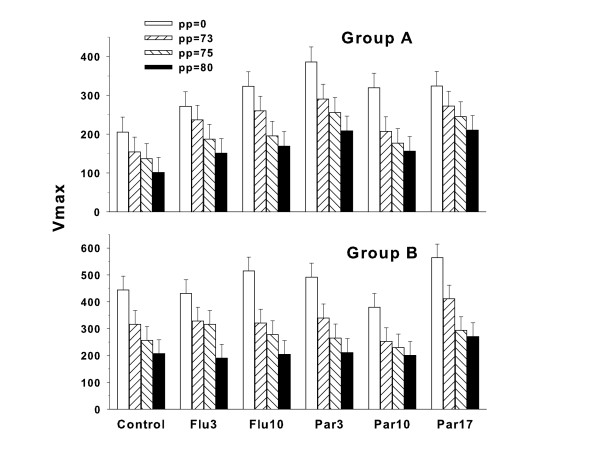
**Acoustic startle/PPI: Least square Mean (± SEM) startle amplitude (V**_**max**_**) measured in units of voltage change (mV)**. Group A was tested on P59 and Group B on P124. No treatment group effects were obtained for either Group A or B, nor any treatment × prepulse interactions. Prepulse was significant and showed that the greater the prepulse intensity the greater the inhibition of the startle response. Number of litters in Group A = 21. Progeny group sizes for Group A: Control = 20, Flu3 = 21, Flu10 = 21, Par3 = 20, Par10 = 21, Par17 = 21. For Group A, 1 Control and 1 Par3 animal had missing data because they were tested with the incorrect startle program. Number of litters for Group B = 20. Progeny group sizes for Group B: Control = 20, Flu3 = 20, Flu10 = 20, Par3 = 19, Par10 = 20, Par17 = 20. For Group B, 1 Par3 animal's data were missing because the test chamber power was not activated.

For the Group A startle response ANOVA, the treatment group main effect was not significant but showed a trend (F(5,98.2) = 1.95, p < 0.10). There was no significant treatment × prepulse interaction. Prepulse intensity was significant (F(3,354) = 100.32, p < 0.0001) showing that prepulse inhibition of ASR was obtained. In the ANCOVA analysis, no significant treatment main effect was seen (F(5,98) = 1.86, p = 0.11). The treatment × PPI interaction was not significant. The prepulse main effect was significant (F(2,236) = 68.01, p < 0.0001). The data are shown in Figure 5**(top panel)**. ES for Group A = 0.27 (small).

**Figure 6:**
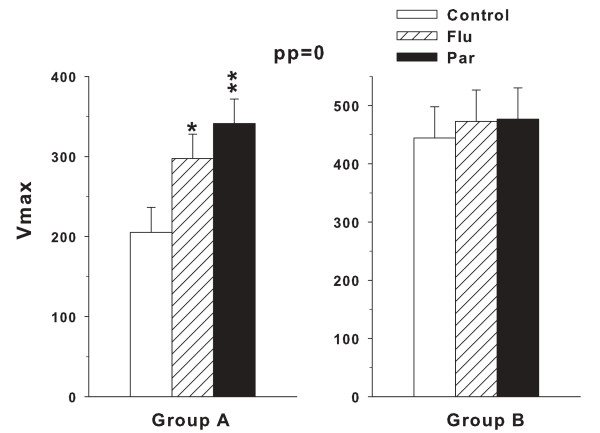
**Acoustic startle for the no-prepulse trials: Data are group least square mean (± SEM) startle amplitude (V**_**max**_**) measured in units of voltage change (mV)**. Group A was tested on P59 and Group B on P124 as for Figure 5. Group sizes are as in Figure 5. *p < 0.05, **p < 0.01 compared with Control.

An inspection of Figure 5 taken together with the treatment main effect trend (p < 0.10) led us to conduct two follow-up analyses. In both analyses we combined the two Flu groups' data and the three Par groups' data into single Flu and Par groups. In the first follow-up analysis, all prepulse trials were included in a treatment × prepulse ANOVA. The treatment main effect (F(2,39) = 7.13, p < 0.003) and prepulse main effect (F(3,177) = 88.43, p < 0.0001) were both significant and the treatment × prepulse interaction showed a trend (F(6,177) = 1.89, p < 0.09). Post hoc group comparisons (averaged across prepulse intensities) showed that both the Flu and Par groups differed from Control (mean ± SEM: Control: 149.0 ± 23.5; Flu: 224.4 ± 23.0; Par: 252.7 ± 23.0). This facilitated startle effect was most pronounced on the no-prepulse trials, therefore, the second follow-up ANOVA was with the combined dose groups but only on the no-prepulse trials. ES for the latter = 0.44 (medium). With no prepulses in the analysis, the ANOVA was a simple one-way analysis with 3 groups. The treatment effect was significant (F(2,39) = 6.55, p < 0.004). This effect is shown in Figure 6**(left panel)**. Post hoc group comparisons revealed that both drug groups showed significantly increased startle amplitude compared with the Control group. ES for the latter = 0.42 (medium).

For Group B the treatment × prepulse ANOVA showed no significant main effect of treatment group or treatment × prepulse intensity interactions. Prepulse intensity was significant (F(3,339) = 92.60, p < 0.0001) showing that ASR was significantly modified by the PPI procedure (Figure 5, **bottom panel**). The ANCOVA analysis showed a similar outcome, no significant treatment main effect or treatment × prepulse interaction. The prepulse main effect was significant (F(2,226) = 48.20, p < 0.0001). ES = 0.18 (small).

As for Group A, follow-up analyses with dose-levels of each group combined were conducted for Group B, and there was no significant treatment main effect or treatment × prepulse interaction. ES = 0.06 (small). Prepulse was significant (F3,171) = 72.39, p < 0.0001). An additional analysis performed on the no-prepulse trials by one-way ANOVA showed no significant treatment effect (Figure 6, **right panel)**. ES = 0.06 (small).

### Porsolt FST

**Figure 7:**
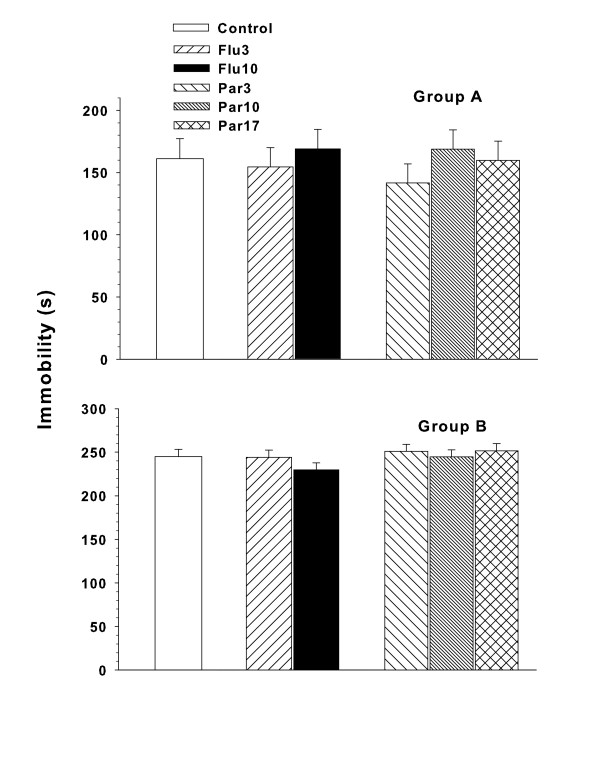
**Porsolt forced swim test: Least square Mean (± SEM) immobility time on the test trial (day-2; out of 300 s)**. Animals received a 15 min training trial 24 h previously. Group A was tested on P61-62 and Group B on P126-127. Group sizes: Number of litters tested in Group A = 21; data are based on the following number of subjects/group: Control = 19, Flu3 = 21, Flu10 = 20, Par3 = 21, Par10 = 21, Par17 = 21. Two Control and one Flu10 animal had missing data tracks on the DVD because of playback issues. Number of litters tested in Group B = 20; data are based on the following number of subjects/group: Control = 19, Flu3 = 19, Flu10 = 20, Par3 = 20, Par10 = 20, Par17 = 20. Tracks for two Control and one Flu3 animal would not playback on the DVD.

For Group A, there were no significant treatment group effects found on immobility time (Figure 7, **top panel**) or latency to immobility (not shown) in the FST. ES = 0.20 (small).

Similarly, for Group B there were no significant effects of treatment group found on immobility time (Figure 7, **bottom panel**) or latency to immobility (not shown) in the FST. ES = 0.21 (small).

### Corticosterone

**Figure 8:**
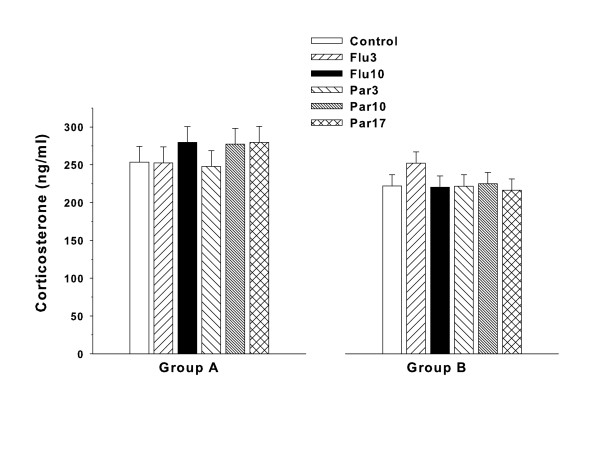
**Corticosterone: Mean (± SEM) plasma corticosterone levels obtained immediately after the end of the FST**. No differences were observed for either drug compared with Control. Group sizes: Number of litters tested in Group A = 21; data are based on the following number of subjects/group: Control = 21, Flu3 = 21, Flu10 = 21, Par3 = 21, Par10 = 21, Par17 = 21. Number of litters tested in Group B = 20; data are based on the following number of subjects/group: Control = 19, Flu3 = 19, Flu10 = 19, Par3 = 19, Par10 = 19, Par17 = 19.

For Group A, all groups showed high corticosterone levels at the end of the 5 min FST compared with typical basal levels (~50 ng/ml) however there were no treatment differences among the groups (Figure 8, **left panel**).

For Group B, again all groups showed increased corticosterone levels in response to the stress at the end of the FST but there were no significant effects of treatment obtained (Figure 8, **right panel**).

## Discussion

Fluoxetine, paroxetine, and many other SSRIs have proven to be efficacious for the treatment of adults with depression, premenstrual syndrome, obsessive compulsive behavior, panic disorder, generalized anxiety disorder, social anxiety disorder, and post-traumatic stress disorder [1-3]. These drugs have been found to be safer and have fewer side-effects than tricyclic antidepressants. SSRIs have also been used in children and adolescents, and fluoxetine is approved for use in those 6-18 years of age. In children whose nervous system is still developing, there are concerns about the long-term effects of these drugs.

In a recent preclinical experiment examining paroxetine and fluvoxamine, it was reported that these drugs reduced time-in-open in an EPM [11]. Decreased time in open in the EPM is the most widely accepted index for this test of increased anxiety-like behavior [23]. In the experiment by deJong et al. [11] this change occurred for both drugs 20-30 days after the end of treatment. No change was seen in the number of zone crossings. These authors also tested the animals in an elevated T-maze (the EPM was used with one arm blocked). Out of 5 trials for time to enter an open arm after being placed in a closed arm, both SSRI-treated groups entered an open arm faster on trial-2 compared with controls but not on trial-1 or 3-5. When the situation was reversed and they were given an additional trial by being placed in an open arm and timed for entry into a closed arm, no differences were found. No differences in ASR/PPI were obtained nor were changes obtained on immobility time in the FST.

We sought to further test the effects of adolescent exposure to SSRIs and followed many of the design features used previously [11], except using more dose levels of each drug and including separate groups of animals, half tested while on-drug (Group A) and half tested off-drug (Group B; 60 days post-treatment). If long-term effects of SSRI treatment were obtained, it would potentially represent a concern for the safety of the drugs.

Both drugs produced body weight reductions at the highest doses tested by P44, i.e., after 12 days of treatment, and these reductions remained through the end of treatment (P62). But the effects showed recovery to non-significant differences one week post-treatment. Behaviorally, no treatment-related effects on the EPM were obtained on the principal measure of anxiety, i.e., time-in-open. In addition, no differences were found for number of zone crossings or latency to first open arm entry. The Group A Par17 animals showed a significant reduction in head-dips, an effect seen in no other group, however this effect was not seen in Group B when tested 60 days after drug cessation. deJong et al. [11] did not report head-dips, therefore, it is not possible to make a direct comparison on this variable, however, a reduction in head-dips is generally interpreted as an increase in anxiety [24], although it is an index of anxiety that is secondary to time-in-open and therefore provides less persuasive evidence of a significant change in anxiety. However, this effect would be consistent with the finding of deJong et al. [11]. A recent study showed that rats treated from P25-49 with 12 mg/kg of fluoxetine had reduced time-in-open in the EPM [25] 7 days post-treatment, a finding consistent with reduced head-dips that we found while animals were on drug. It is unclear why adolescent exposed rats and mice show greater inconsistency in EPM responses to SSRIs than adult rodents. Some of it may be related to the different exposure ages as there is no consistent definition of adolescence in rodents. Doses and routes of drug administration as well as duration of treatment and end of treat to test interval vary widely across studies, increasing the difficulty of discerning patterns.

Neither we nor deJong et al. (2006) found any effect on the FST test of swimming despair [17,18] and this finding is different than that reported by Homberg et al. [25] but they treated rats earlier (P25-46); moreover, they found no differences in rats treated at a later age. Other studies have found opposite or paradoxical effects of adolescent exposure to fluoxetine in mice, but only in one of the two strains tested [26]. To the extent that the FST is a valid preclinical test of depression, the data do not suggest that adolescent exposure to fluoxetine or paroxetine result in long-term changes in immobility. This is consistent with a newer report of chronic fluoxetine treatment in mice from P14-42 showing no FST effects [26] and the data cited above [12]. We scored the FST for immobility time as originally described by Porsolt et al. [17,18]. We did not score the test using the recently suggested indices of active swimming and wall climbing [20]. While we cannot rule-out the possibility that these additional indices might have uncovered other effects, we did not include them because our interest was not differentiating among different classes of SSRIs, which was the reason these measures were introduced, but rather whether immobility was a long-term consequence of drug exposure after adolescent treatment.

Others have administered SSRIs by osmotic pump in order to maintain plasma concentrations in animals within the same range as human therapeutic concentrations [27,28]. The intent of these studies was different than ours. These studies were designed to assess the molecular targets of SSRIs. For this purpose maintaining a constant drug concentration is desirable but this goal was not necessary for our purposes of determining if these drugs have persistent long-term effects long after cessation.

deJong et al. [11] reported no changes in ASR/PPI. We replicated their parameters and in agreement, observed no changes in ASR or PPI from either drug 60 days post-treatment. However, while on-drug, we saw a trend toward ASR facilitation that did not interact with PPI. To further explore the data, we conducted follow-up analyses by combining the two fluoxetine groups into one pooled group by averaging the data for the fluoxetine animals in each litter together to create a single, merged group. We did the same for the paroxetine, merging the data from all three dose levels together among littermates to create a single paroxetine group. Two follow-up analyses were performed. These were: (1) with all prepulse trials included and (2) with no-prepulse trials included, i.e., only the unmodified, basic ASR trials. Both analyses resulted in the same finding: the pooled fluoxetine and pooled paroxetine groups exhibited increased ASR amplitude compared with Controls. By contrast, when Group B ASR-PPI data were analyzed the same way, no residual effect or trend toward an effect was observed. This is in contrast to a study that found that P24-46 fluoxetine reduced ASR at a higher dose than we used (12 mg/kg), but similar to our data, found no effects at later ages [25].

The present data should be viewed within the limitations of the study. As in the deJong et al. [11] and Norcross et al. [12] studies, we tested only males. It may be worthwhile to test females as many drugs exhibit sexually dimorphic responses. It is also worth considering that future experiments might test a broader range of doses in order to more thoroughly test for possible adverse effects. Finally, other behavioral tests might be worth considering; Norcross et al. [12] also included open-field and cued fear conditioning with extinction, but even more tests might be considered such as fear-induced acoustic startle facilitation. There are many tests of anxiety, conditioned fear, sensorimotor gating, and depression, of which only a subset were used here or in the other studies cited that tested for residual effects. A more extensive battery of tests might reveal effects not detected herein.

Overall, the data show that for the doses of fluoxetine and paroxetine tested by an oral route during adolescent to early adult brain development (operationally defined as P33-62), caused minor and only transient reductions in body weight gain, a small but significant ASR facilitation during treatment that did not remain 60 days post-treatment, and a high-dose only (Par17) reduction in EPM head-dips while on-drug but not off-drug. The findings, when considered in terms of the power to detect differences in the present experimental design (≥ 20 animals per group), multiple dose levels, use of a within-litter design that improves subject matching, and inclusion of both on- and off-drug cohorts, suggest that there is no signal of adverse effects present in the data that might raise concern over the long-term safety of the drugs when treatment is during an interval spanning adolescent brain development in rats, in agreement with previous findings in mice [12]. It was recently reported that fluoxetine given by continuous infusion via minipumps from P14-42 results in anxiogenic effects in mice while on-drug on tests of novelty-induced hypophagia in Swiss-Webster (SW) and C57BL/6 Charles River (B6) mice, however there were no effects in the EPM in the SW strain and reduced time-in-open in the B6 strain but no effects on open-field center time or FST in either strain. These effects disappeared off-drug [26] which is entirely consistent with the present findings. Thus, while SSRIs induce relatively reliable effects on-drug in adult rodents, their effects from adolescent exposure are less consistent. Our data are consistent in that we saw only small effects while on drug and no long-term effects long after drug discontinuation. The latter provides some evidence that these drugs are not neurotoxic when given during adolescent stages of brain development at least on the behavioral indices used here.

## Financial competing interests

The research reported herein was supported by a research contract to Cincinnati Children's Research Foundation (CCRF) under CCRF required provisions of academic freedom from sponsor influence, viz., that work performed under the auspices of the CCRF are under the sole discretion of CCRF and the investigators. Under these provisions, the principal investigators are free to publish all results as they deem scientifically appropriate. In addition, the CCRF authors have received no reimbursements, fees, salary, honoraria, or other benefits from the sponsor. Dr. Morford is an employee of Eli Lilly and Company. The article processing charge was paid by CCRF. The CCRF authors declare that they have no stock or other financial interest in the sponsoring company and stand to make no financial gain or loss from the publication of these data now or in the future. The CCRF authors hold no patent now or in the future related to this manuscript. The authors have received no reimbursements, fees, funding, or salary from an organization that holds or has applied for patents relating to the content of the manuscript. The CCRF authors declare no financial conflicts of interest.

## Non-financial competing interests

The authors declare no non-financial competing interests (political, personal, religious, ideological, academic, intellectual, commercial, or any other) in relation to this manuscript.

## Authors' contributions

CVV was Principal Investigator, participated in the design, data analyses, and interpretation of the data, oversaw the project, and wrote the manuscript. LRM participated in the design and provided on-sight quality assurance inspections at regular intervals to ensure protocol compliance. DLG conducted the corticosterone assays. MRS was in charge of study blinding of animals to ensure that technicians had no knowledge of treatment groups, made coded drug solutions, and provided drug solutions to ABC Laboratories blinded for potency and stability assays on drug solutions. MTW was Co-Principal Investigator, participated in the design, data analyses, and interpretation of the data, and contributed to the manuscript. All authors have read and approved the final manuscript.
